# The Charité Hospital, Paris

**Published:** 1907-11-16

**Authors:** 


### THE EVOLUTION OF MEDICINE.

### THE CHARITE HOSPITAL, PARIS.

Modern needs compel the demolition of the Charité Hospital at Paris. The hospital was established and was served by the brethren of Saint Jean de Dieu, whose last provincial chapter in 1789 was rendered eventful by the delivery of a *lettre de cachet* against the Provincial Father Ignace-Joubin Desmatières, the last letter signed by Louis XVI.

Jean de Dieu was a Portuguese saint (1495-1550) who busied himself in nursing the sick poor, and the brethren who followed his teaching afterwards possessed many houses in Europe, and had travelled as far as America, though even as late as 1601 they had no establishment in France. In that year Marie de Medicis, who had learnt in Italy to value their labours, summoned Brother Jean Bonelli and four others from Florence, and placed them in the faubourg Saint-Antoine under the title of "Religieux de la Charité." But they only stayed near the Seine for a short time, as Queen Margaret moved them to a little house surrounded with a large garden close to the abbey of Saint-Germain-des-Près. In the following year, 1602, they received letters patent from Henri IV. according them many valuable privileges, which were afterwards confirmed by Louis XIII. and Louis XIV. From the beginning men alone were received into the hospital, and in 1723 it contained 172 beds, a number which was afterwards increased to 250 by the liberality of Cardinal Richelieu, who built a large ward known until 1870, when it was called the Velpeau ward, as the Virgin ward.

The brethren were 70 in number. The hospital contained four wards, one for those suffering from severe fevers, a second for surgical cases, a third for those who were only ailing and for those who needed a few days' rest, whilst the fourth was set aside for patients with stone. The brethren rose at four in the morning both in winter and summer, and went to the church. Service over, they visited the wards, and distributed the first meal of soup, made the beds of the patients, and, after dressing them, attended to the sick and needy who came as out-patients. At six the physician made his round, attended by the ward-master, the surgeon, and the apothecary—these three always being brethren of the house. The ward-master showed the patient to the physician, whilst the surgeon and the apothecary entered the directions for treatment each in his own book.

The patients had an hour's rest after the physician's visit, and prayers were then said aloud in each ward, after which Mass was celebrated. On a given signal dinner was served, and the brethren returned to the wards singing the *Laudate Dominum* or the *Miserere* as they distributed the food. Between dinner and supper the patients were instructed in the Christian doctrine or they were read to by the monks. At supper time the brethren again brought in the food, the Superior said grace, and the ward-master distributed the meal, after which the Superior prayed before the altar in the ward and sang the *Salve Regina*. He then left in procession with the cross and lighted candles, and went with the brethren through each ward to give holy water to the patients, singing the while the *Miserere* and other Psalms. On their return the procession sang the *Memorare* of St. Bernard and the *De profundis*; the brethren then went back to the wards, made the beds of the patients, attended to their wants, and left them for the night.

The brethren, however, were more than mere sick nurses, for they applied themselves to anatomy, surgery, pharmacy, chemistry, and botany. They made careful observations of the cases which came under their care, and performed all the usual surgical operations then in vogue. Thus they trephined, cut for stone, opened empyemata, relieved strangulated herniæ, cured aneurysms, and amputated limbs. It is hardly surprising, therefore, they were not viewed with very friendly eyes by the Corporation of the Master Surgeons of Paris, who haled them before the Parliament in 1721.

The brethren were wise in their generation, for they appointed from time to time one of the recognised surgeons of Paris to help them, and they admitted to their wards the apprentices of the barber surgeons. Boyer (1757-1833), even when he was a Baron of the Empire, never forgot that his first insight into surgery was gained in this humble manner, and he would often recall the debt he owed to his friend and old master, Brother Potentien of La Charité. The hospital was fortunate in possessing an annexe or convalescent home, founded in the Rue de Bac in 1652. It received twelve convalescents every week from the parent house, who were attended by four brethren. The convalescents were allowed to go out daily to attend to their business or to look for work. The expenses of the house were defrayed by Angelica de Faure, a great lady of the time, who chose to remain anonymous until her death.

